# Review: Improving quality of life in patients with differentiated thyroid cancer

**DOI:** 10.3389/fonc.2023.1032581

**Published:** 2023-01-27

**Authors:** Pia Pace-Asciak, Jonathon O. Russell, Ralph P. Tufano

**Affiliations:** ^1^ Department of Otolaryngology – Head and Neck Surgery, University of Toronto, Toronto, ON, Canada; ^2^ Department of Otolaryngology – Head and Neck Surgery, Johns Hopkins University, Baltimore, MD, United States; ^3^ Department of Otolaryngology - Head and Neck Surgery, Sarasota Memorial Health Care System Multidisciplinary Thyroid and Parathyroid Center, Sarasota, FL, United States

**Keywords:** thyroid cancer, quality of life, surgery, radiofrequency ablation, active surveillance

## Abstract

Well differentiated thyroid cancer is a common malignancy diagnosed in young patients. The prognosis tends to be excellent, so years of survivorship is expected with low risk disease. When making treatment decisions, physicians should consider long-term quality of life outcomes when guiding patients. The implications for treating indolent, slow growing tumors are immense and warrant careful consideration for the functioning years ahead. Surgery is the standard of care for most patients, however for a subset of patients, active surveillance is appropriate. For those wishing to treat their cancer in a more active way, novel remote access approaches have emerged to avoid a cervical incision. In the era of “doing less”, options have further expanded to include minimally invasive approaches, such as radiofrequency ablation that avoids an incision, time off work, a general anesthetic, and the possibility of post-treatment hypothyroidism. In this narrative review, we examine the health related quality of life effects that surgery has on patients with thyroid cancer, including some of the newer innovations that have been developed to address patient concerns. We also review the impact that less aggressive treatment has on patient care and overall wellbeing in terms of active surveillance, reduced doses of radioactive iodine (RAI) treatment, or minimally invasive techniques such as radiofrequency ablation (RFA) for low risk thyroid disease.

## Introduction

Well differentiated thyroid cancer (WDTC) is one of the most common neck malignancies in patients under 40 years of age (1). A large number of cancer survivors exist due to the rising incidence of diagnosis at a young age and the excellent long-term survival rate post-surgery. Even though the prognosis is excellent, and the 10 year survival rate exceeds 90%, sometimes the treatment can leave patients with a sequelae that affects their quality of life (1). Upon diagnosis, patients are often counselled that they have a slow growing, indolent cancer with an excellent prognosis. While this holds true for the majority of patients, this doesn’t account for the physical and emotional burden that can occur post-surgery, impairing the ability to socialize or work.

The implications of thyroid cancer treatment are immense and warrant a careful look at the long-term effect on the quality of life in addition to the projected prognosis. Studies have shown that the health related quality of life (HRQOL) of thyroid cancer survivors can be negatively affected for up to 20 years after treatment (2). With the rising number of cases in patients under 45, years of surveillance are required post treatment to monitor for recurrence decades later, to adjust the dose of lifelong thyroid replacement medications, to monitor for side effects of radioactive iodine treatment (RAI) or to address any functional impairment post-surgery such as dysphonia or dysphagia.

There is no one definition of quality of life (QOL). The World Health Organization defines quality of life (QOL) as an individual’s perception and expectation of a certain standard of excellence as regards to his/her social and cultural environment (3). This term tends to be multidimensional and is affected by numerous factors such as physical health, psychological state, level of independence, social relationships and is associated with the environment which the person is living in (4). Assessing the QOL post-treatment is an important aspect of cancer care that deserves as much attention as immediate treatment of the disease itself. This aspect of cancer care tends to be more difficult to quantify, however various validated questionnaires have attempted to capture this data. It has been shown that QOL in cancer patients is not predicted by prognosis alone and QOL in thyroid cancer survivors can be worse than in patients with other malignancies who may have a worse prognosis (5). Gamper et al. reinforce the idea that functional impairment related to treatment for thyroid cancer is unrelated to the favorable clinical outcome (6).

Over the past few years there has been an increased focus on the HRQOL of patients with thyroid cancer. This is in response to the relative paucity of data, the years of disease free survival, and a shift towards “doing less”. Given that patients with thyroid cancer tend to have a life expectancy that does not impair their survival, ensuring their HRQOL during surveillance plays an important role in their care. In this narrative review, we collate the data on the HRQOL effects of surgery in patients with thyroid cancer, including some of the newer innovations that have been developed to address patient concerns. We also review the impact that less aggressive treatment has on patient care and overall wellbeing in terms of active surveillance, reduced doses of radioactive iodine (RAI) treatment, or minimally invasive techniques such as radiofrequency ablation (RFA) for low risk thyroid disease.

### Surgery

Knowledge that thyroid carcinoma is part of a clinical spectrum of low, intermediate and high risk disease is an important aspect for guiding patients through their options for surgery, active surveillance or thermal ablation. Aggressive disease is met with aggressive resection resulting in total thyroidectomy, +/central neck dissection, and even lateral selective neck dissection when warranted, followed by post-operative lifelong levothyroxine replacement, radioactive iodine and external beam radiation in the advanced disease state (1). For the most part, there has been a shift toward less aggressive surgical intervention, reduced RAI use, and less thyroid hormone suppression for lower risk disease (well differentiated thyroid cancer > 1 cm but < 4 cm without extrathyroidal extension, or clinical evidence of cervical lymphadenopathy (cN0) (1). As clinicians, it can be challenging to guide patient decision-making while balancing the risks of disease recurrence and interventions that may affect the long-term quality of life. Most of the time, surgical resection of low risk carcinoma with clear margins leads to cure (7), however complications can and do exist. Even though select patients demonstrate equal oncological control of their cancer with total thyroidectomy or lobectomy, factoring in their quality of life post-surgery is an important determinant to consider when discussing the extent of surgery with patients (8–11).

Completeness of surgical resection is the most important determinant of outcome whereas treatments such as RAI treatment and TSH suppression are reserved as adjuncts to cancer control (8, 9). How much or how little to surgically remove is an important consideration when achieving a favorable long-term outcome that needs to be balanced with an overall well-being. A handful of articles have examined whether there is a difference in the QOL of patients receiving a thyroid lobectomy compared with total thyroidectomy (12, 13) and the results are conflicting. Malterling et al. compared the short and long-term outcomes as well as the quality of life in Swedish patients with papillary and follicular cancer after treatment with total thyroidectomy and post-operative RAI (77 patients) compared with treatment with subtotal thyroidectomy and occasional RAI (53 patients) (12). Overall, no significant difference was found in outcome between the two groups in the 10 year cancer-specific survival rate irrespective of stage. Specifically, aggressive treatment for TNM stage I-II did not confer an advantage and led to more unwanted side effects. The quality of life was assessed by questionnaire 8 to 22 years after their primary operation (88% response rate) and found 7 patients lived with permanent complications including RLN palsy, hypocalcemia and ongoing cancer, or recurrence, however a difference in the quality of life was not appreciated due to the intervention received. No significant difference in mental and physical health was found on SF-36 questionnaire in age- and sex-matched Swedish reference population compared with thyroid cancer patients. This study is limited by a small number and potential bias from years after surgery.

In a larger based study which included 1005 patients with differentiated thyroid cancer, Nickel B. et al, found health related QOL issues in 775 patients (77.1%) after diagnosis and treatment of their cancer (median time between diagnosis and interview was 23.1 weeks (range, 1.9 – 90.9 weeks)) (13). Papillary thyroid cancer was the most common diagnosis (889 of 1003, 88.6%), where roughly half of patients had tumors less than 2 cm in size (564 of 1000, 56.4%), and 791 patients received total thyroidectomy (78.7%). Those patients who had a total thyroidectomy (without neck dissection) were 1.5 times (odds ratio, 1.49; 95% CI, 1.04-2.12) more likely to report health related QOL issue or an adverse effect of treatment compared with patient who underwent hemithyroidectomy. More specifically, the common themes that emerged from patient responses were physical concerns (663, 66.0%), psychological (187, 18.6%), lifestyle (82, 8.2%), or no issue or adverse effect (246, 24.5%). High or very high psychological distress scores (Kessler Psychological Distress Scale) were found compared with the national figures (Australia) (20.6% vs.11.7%). Examples of the most common physical concerns included; fatigue, low energy levels, voice concerns, difficulties titrating medical levels (13). Other studies have found similar concerns as Nickel B et al, demonstrating that the quality of life issues relevant to thyroid cancer patients cross culturally include fatigue and exhaustion, quality of sleep, employment, social support, fear of cancer progression, fear of second operation, difficulties swallowing and globus sensation (14). Worldwide, the most common theme in thyroid cancer survivors include fatigue and sleep issues (6, 15–17).

Various studies have demonstrated a significant negative impact on patient’s QOL when a visible scar is present from a midline anterior neck incision (18, 19). This seems to be more of a cultural phenomenon, and often placement of the surgical scar within an existing natural skin crease can hide the scar nicely, resulting adequate patient satisfaction in most parts of the world (20). Regardless, novel approaches for accessing the thyroid remotely are gaining popularity in certain parts of the world to avoid a visible scar that the traditional surgical approach requires for gland removal. Choi et al. used a Dermatology Life Quality Index (DLQI) to investigate the impact of thyroid scars on the quality of life and found the mean score to be 9.02, similar to that of patients suffering from chronic skin diseases such as psoriasis, vitiligo and severe atopic dermatitis (19). Various remote access techniques have been developed to hide a cutaneous scar in the anterior neck to less visible locations such as under the breast or in the axilla, and more recently intraorally. The transoral endoscopic thyroidectomy vestibular approach (TOETVA) for removal of the thyroid has become a favored approach since it allows the surgeon midline access to the thyroid with little soft tissue dissection, no cutaneous scar, and safe same-day discharge with minimal discomfort for low risk thyroid disease (21–29). Although the operative time is longer with TOETVA, and the costs are greater with remote access techniques, in certain cultures, these are acceptable trade-offs to avoid a visible scar. When the DLQI was applied in the early post-operative period after receiving the transoral vestibular approach, the median score was 3, a significantly favorable outcome compared with an open approach (26). Furthermore, no significant differences in the incidence of major complications between TOETVA and transcervical thyroidectomy (1.5% vs 2.1%, p=0.75) were noted for both benign and low risk malignancy, making it a sound approach in select patients concerned about a visible scar (29, 30). Other reports have demonstrated the safety and efficacy of remote access techniques for eliminating a visible scar (31).

In a retrospective study by Sun H et al., patients with clinically positive central neck nodes and papillary thyroid cancer with tumor sizes < 1.5 cm in the upper pole and < 3 cm in other parts of the thyroid underwent total thyroidectomy with central neck dissection *via* TOETVA compared with open approach by a single surgeon. After propensity score matching, 28 patients underwent TOETVA and 56 patients were included in the conventional open thyroidectomy group (32). In term of oncological control, the mean number of retrieved central lymph nodes (p=0.202), metastatic central lymph nodes (p=0.421), and Tg level without thyroid-stimulating hormone (TSH) stimulation (p=0.686) 3 to 54 months after surgery did not differ between the TOETVA or open conventional group. At least within the short term, this study demonstrates oncological success. In terms of safety, a significant difference was not noted between the rate of transient vocal cord palsy (p=1.000), or transient hypoparathyroidism (p=0.870) between the two groups.

With both TOETVA and the open conventional approach, complications such as permanent recurrent laryngeal nerve (RLN) palsy or permanent hypoparathyroidism are rare but do occur with surgery. For high-volume thyroid surgeons, the open neck approach can result in less than 1% of permanent RLN injury in primary cases, with up to 30% of impaired RLN function after revision thyroid surgery (33, 34). Most of the literature shows comparable rates to the standard open thyroidectomy with no significant difference in safety when the procedure is done with an endoscope or *via* the open approach (32, 35). These numbers are less with thermal ablation for various reasons which include careful patient selection for more anterior based tumors away from the “danger triangle”, and low risk carcinomas. The cornerstone of therapy is based on the preoperative decision making which involves balancing the risks and benefits of surgery or any treatment at all. Impaired vocal fold mobility can impact a patient’s ability to work or socialize, can result in breathing difficulties in up to 75% of patients during activity, dysphagia in up to 56%, aspiration in as many as 44% of patients creating significant impact to their quality of life (34). Thus, intraoperative nerve monitoring has played an important role for guiding favorable outcomes particularly for advanced malignancy, re-operative surgery or anticipated aberrant anatomy (36).

TOETVA has a few potential complications worth mentioning that do not exist with the open convention approach. For example, transient mental nerve injury can occur during placement of the endoscopic port in the inferior vestibule. In a recent systematic review, mental nerve injury was 5.8% (102/1,887 patients), whereas other reviews report a prevalence ranging 1-5% (35, 37). Skin bruising and dimpling of flap perforation are rare complications with TOETVA, however can be disappointing to a patient who opted for a scarless approach. Another rare (2 out of 1887 patients) but potentially serious complication is a carbon dioxide embolism caused by prolonged high insufflation pressures or a tear in the vessel promoting entrance of C02 into the circulation (35). Other insufflation-related adverse events include pneumomediastinum, pneumothorax or excessive hypercarbia. Gasless TOETVA has also been invented to avoid such complications (38). The literature also describes the unique but rare risk of delayed tracheal rupture caused by accidental dissection, surgical needle puncture, Hegar dilation, trocar placement or thermal injury from the energy device (39). Through careful blunt dissection and use of the energy device these complications can be mitigated.

Chronic hypoparathyroidism post-total thyroidectomy can result in persistent hypocalcemia due to low circulating parathyroid hormone from devascularized or resected parathyroid glands. Symptoms of hypoparathyroidism have been shown to have negative effects across several domains (40). In a questionnaire to 264 hypoparathyroid patients post-thyroid surgery, Buttner et al, found significant impairments in social, physical, cognitive and emotional functioning leading to an impaired quality of life (41). In response to this complication, certain centers are incorporating parathyroid autofluorescence into their practice as a means for improving the detection of these glands over visualization alone (30).

Patients after total thyroidectomy for malignancy require life-long thyroxine replacement and regular evaluation of TSH serum levels as well as thyroglobulin to screen for recurrence. These simple blood tests are an easy approach for biochemical surveillance. However, a subset of patients experience chronic fatigue syndrome known as asthenia after total thyroidectomy (TT), even if their TSH is within the normal range. In a prospective observational cohort study that included 182 patients, Luddy et al. found that 42% of patients had asthenia after total thyroidectomy (TT) compared with 4% after thyroid lobectomy (TL) on Brief Fatigue Inventory questionnaire more than one year post surgery (42). Patients were also more likely to have asthenia if they had surgery for a malignancy compared with benign disease, with an odds ratio of 10.4 (95% CI 3.86-28.16) when TT was compared to TL for patients with malignancy compared to benign disease (2.05, 95% CI 1.17-3.61) (42). Although the rate of post-operative hypothyroidism after a lobectomy alone varies considerably in the literature, doing less for low risk disease may improve long-term outcome in the correct clinical scenario.

### Radioactive iodine treatment

Radioactive iodine (RAI) has been the standard treatment post-thyroidectomy for papillary and follicular thyroid carcinoma for decades (1). Even though RAI administration is relatively routine, it is fraught with early and late side effects, which can impact chewing, speech, taste, saliva, anxiety levels including the risk of a second malignancy (43). Treatment with RAI also delays family planning by a year which can be of concern for women in their prime reproductive years (43). Although the treatment itself has unwanted effects, the side effect with the most impact is the period prior to RAI treatment which requires patients to be rendered hypothyroid, so uptake of RAI was more avid. In the past, the pre-ablation period would lead to significant physical and emotional instability and reduce the QOL in patients during and after treatment as they try to bring their TSH into the normal range. Since the development of recombinant human thyroid stimulating hormone (rhTSH) (also known as thyrogen), this practice has become obsolete, making the process more tolerable. Thus, rhTSH has significantly improved the aftercare post-thyroidectomy as an alternative for thyroid hormone withdrawal pre-RAI ablation.

Post-operative treatment with RAI for low risk well differentiated thyroid cancer is controversial, and has been shown repeatedly that it is unlikely to have any meaningful benefit for papillary microcarcinoma or patients with low risk disease that has no other worrisome features (1, 44–48). Even the dose of RAI has been questioned, leading the American Thyroid Association guidelines to recommend use of a lower dose of RAI in low and intermediate risk disease (1, 49). Studies have shown that the use of high dose RAI (ie.100 mCi) has no advantage for remnant ablation over a lower dose of 30-50 mCi for low to intermediate risk disease (1, 49). Mazzaferri et al. examined the effects of more than 1000 patients treated with RAI following thyroidectomy for WDTC and found a decrease in cancer-related deaths and recurrences in patients who were older than 40 years or who had isolated tumors larger than 1.5 cm compared to those who did not receive RAI (50). Post-operative RAI plays a role in certain clinical scenarios, however in the setting of low risk disease, it’s role is minimal in reducing recurrence of thyroid carcinoma.

In a cross-sectional study, Almeida et al. found that patients who received a higher dose of 150 mCi of RAI had significantly worse pain, swallowing, chewing, speech, taste, and anxiety (51). Similarly, Ahn et al. found a decrease in the QOL in patients that were treated with total thyroidectomy (TT) and RAI compared with TT alone (52). Even when a lower dose (median dose was 80 mCi) of RAI was used, patients experience lower scores in QOL measurements, despite matching for TSH, and levels being in the normal range (52). This accumulating evidence has resulted in most North American institutions moving away from treating patients with RAI for low risk disease such as PTMC, or performing total thyroidectomy unless more concerning high risk features are present. In the era of “doing less”, selective and judicious administration of RAI can translate to improved well-being post treatment.

### Active surveillance

The push to be conservative is based on the notion that “doing less” can lead to a better QOL. However, this warrants a close look at both sides of the issue so the impact imposed by the numerous repeat ultrasounds, biopsies, and bloodwork to actively surveil the patient as well as the emotional and psychological aspects of living with a known cancer is assessed. However, most of the current literature demonstrates significant patient satisfaction in a select group of patients wishing to avoid surgical intervention.

Nakamura et al. compared the quality of life and psychological impact of patients with PTMC who were under active surveillance (AS) and those who underwent immediate surgery (53). In a cross-sectional study, 347 patients with low risk PTMC who underwent AS (n = 298) or immediate surgery (n=49) completed two questionnaires (thyroid cancer-specific health-related QOL (THYCA-QoL) and the Hospital Anxiety and Depression Scale (HADS)) and the results between the two study groups were compared. In the immediate surgery group, the THYCA-QoL questionnaire revealed more complaints about “voice” (P<0.001), “psychological” (P=0.025), “problems with scar” (P<0.001) and “gained weight” (P=0.047) than the AS group. In the HADS questionnaire, the AS group had significantly better anxiety (P=0.020), depression (P=0.027), and total scores (P=0.014) than the immediate surgery group. Similar findings have been reported in the literature from patients having undergone immediate surgery compared with AS in terms of fatigue, change of voice and appearance, level of satisfaction with similar self-assessed financial burden (54). Although follow up was for a median of 8 months, studies with longer follow-up periods would be beneficial given that patients tend to live for years with little effect on their survivorship. It may also be that there exists a selection bias in studies such as this.

Using a larger population base, Moon et al. investigated the initial treatment choice on their 2-year QOL in patients with low-risk PTMC (55). In a multicenter prospective cohort study on AS (n=674) of their PTMC compared with those that chose immediate surgery (n=381), including lobectomy or total thyroidectomy, the 2-year QOL was best for patients with the least invasive treatments (n=500 for AS, n=238 lobectomy, n = 79 total thyroidectomy). Of the 674 patients, 101 switched from AS to surgery during the follow up period. Thirty five of those patients switched treatment to surgery due to disease progression and were found to have a better QOL on questionnaire compared to the 66 subjects that had no disease progression (55). Jeon et al, found similar QOL issues in patients with PTMC that underwent thyroid lobectomy compared with patients who underwent AS. Specific concerns in the thyroid cancer-specific QOL questionnaire demonstrated statistically significant differences between the groups, with greater health related problems in the surgical group in terms of neuromuscular complaints (coef: 4.99 [CI 0.63-10.62], p = 0.0200), throat/mouth problems (coef: 5.28 [CI 0.18-10.38], p=0.043), and scar problems (coef:9.34 [CI 4.38-14.29], p<0.001) (56). Thus, AS is a reasonable option for patients with PTMC who do not wish to endure these possible risks provided they meet the appropriate oncological criteria.

Lubitz et al. looked at patient preferences retrospectively after they had already undergone treatment (most of which had total thyroidectomy), and found that 536 patients of 1546 (35%) would consider the option of AS if this approach was oncologically equivalent (57). The main reason for patients favoring observation was to preserve their quality of life. Thus, if the burden of surveillance or level of worry is not worse than surgical treatment itself, then this approach is sound (58).

### Radiofrequency ablation

Radiofrequency Ablation (RFA) has emerged as one of the common ways to thermally ablate thyroid low risk WDTC. Thermal ablation is a third option for patients who are anxious about leaving their low risk thyroid cancer untreated. Other indications include recurrent lymph node metastases in patients whom have had a prior total thyroidectomy and neck dissection for their papillary thyroid cancer who are deemed high risk for reoperation or who do not wish to have repeat surgery (59). The most robust data exists for RFA of PTMC as a safe and effective alternative treatment to AS or surgery who wish to actively treat their microcarcinoma but in a minimally invasive way. The advantages for thermal ablation is that it avoids surgical incision in the neck, takes less operative time, does not require a general anesthetic, allows patients to return to work and routine life, and does not render patients hypothyroid. Thermal ablation is suitable for low risk thyroid cancer, similar to those patients that would qualify for AS but who would like to actively treat their cancer.

The evidence for improved HRQOL in patients who have undergone RFA for their low-risk PMC is beginning to emerge. Lan Y et al. compared patients who underwent US-guided RFA with those that had surgery (TT or Lobectomy) by using three validated questionnaires (Short Form healthy survey (SF-36), thyroid cancer-specific quality of life, and Fear of Progression Questionnaire-Short Form) (60). The results show that the physical wellbeing of patients in the RFA group was better than in the surgery group, with improvement in certain scores for the lobectomy group over the TT group. Specifically, there were significantly less complaints relating to overall symptoms, “problems related to scarring” and “less interest in sex” scale scores of patients treated with RFA compared with surgery. However, there was no significant differences in FOP-Q-SF questionnaire scores between the two groups looking at physical health or the social family domain (p >0.05).

In patients that have undergone conventional thyroidectomy compared with thermal ablation of their benign thyroid nodules, Jin et al. demonstrate that thermal ablation provides improved patient satisfaction, post-operative quality of life, and a shorter hospital stay (61). These immediate benefits are improved in patients with benign nodules, however, broader oncological control and risk of recurrence are factors that play into QOL with cancer patients. This has created creative solutions in certain clinical situations, where RFA can be combined with surgery to achieve improved patient satisfaction. Yuan et al, retrospectively evaluated the quality of life in patients who underwent thyroid lobectomy for their unilateral papillary thyroid carcinoma and RFA for the contralateral benign nodules compared with lobectomy alone (62). Those patients who underwent lobectomy plus RFA were found to have improved symptoms of anxiety, physiological health, psychological and sensory features that were measured *via* questionnaire six months post treatment. Of note, after 4.2 year follow-up nine patients (6.1%) in the lobectomy plus RFA group and seventeen (11.5%) in the thyroid lobectomy alone group underwent completion thyroidectomy (P = 0.100). Whether the combined RFA and surgery option is a sound long-term oncological option still remains to be determined with larger prospective data.

For the most part, patients are cured of their papillary thyroid cancer with total thyroidectomy +/- neck dissection followed by post-operative radioactive iodine treatment, however up to 15-30% of patients can have local recurrences in the previously operated tissue bed and neck (59). The American Thyroid Association guidelines recommend surgery as the treatment of choice for neck lymphadenopathy, but some patients may not wish to endure the risks of reoperation, are unfit for surgery, or are at great risk for reoperation in the neck due to scarring (1). Ultrasound guided percutaneous thermal ablation in the treatment of cervical lymph node metastasis of recurrent papillary thyroid cancer has been shown to be a safe and effective treatment as well as a reasonable option for locally controlling small lesions (59). In a systematic review, patients who had their lymph node metastasis treated for their recurrent papillary thyroid cancer had significantly decreased lymph node volumes, low thyroglobulin levels, with low complication rates after treatment (59). The complication rate was higher when ablation was done in the central region (12%), with an overall complication rate of 5%. The results from minimally invasive techniques are encouraging, but need to be appropriately framed in a discussion with patients regarding immediate as well as long term oncological control.

For centers that are not equipped with thermal ablation, cheaper readily available options for treating recurrent papillary thyroid cancer in a minimally invasive way include ethanol *via* chemical ablation. The literature demonstrates how US guided ethanol treatment of metastatic lymph nodes is an excellent alternative to surgery, provided there are a limited number of metastasis from their PTC (63, 64).

## Conclusion

Finding the right balance between oncological control and long term QOL remains an ongoing challenge in thyroid cancer patients. Increasingly, treatment has become more individualized in order to maximize the years of disease free survival in young patients. For low risk well- differentiated thyroid cancer, active surveillance, minimally invasive techniques, such as RFA or ethanol ablation, and even remote access techniques such as TOETVA are reasonable options in select patients. In an attempt to “do less”, to improve the quality of life in patients with papillary thyroid cancer, oncological safety should not be compromised. Technological advancements can enhance a patient’s quality of life but further rigorous studies are need to better define the long-term oncological control.

## Author contributions

PP-A did the literature review, manuscript writing, editing, and finalization of the manuscript. JR and RT contributed to the editing and finalization of the manuscript. All authors contributed to the article and approved the submitted version.
